# Trust in Transition: Exploring Changing Trust in Vaccination in the Context of Long Covid in the United Kingdom

**DOI:** 10.1111/hex.70459

**Published:** 2025-10-21

**Authors:** Anna Dowrick, Alice MacLean, Sue Ziebland, Trish Greenhalgh

**Affiliations:** ^1^ University of Oxford Oxford UK; ^2^ University of Stirling Stirling UK

**Keywords:** Covid‐19, futures, innovation, long Covid, narratives, trust, UK, vaccines

## Abstract

**Background:**

New drugs and vaccines usually come with the promise and hope of benefit. We explore stories about the variable and sometimes disappointing effects of Covid‐19 vaccines in the context of post‐Covid‐19 syndrome (‘long Covid’), aiming to understand how people with long Covid made sense of unexpected vaccine responses and how these experiences impacted their trust in vaccination.

**Methods:**

We carried out 33 interviews with people who described both positive and negative unexpected vaccine experiences connected to long Covid.

**Results:**

Trust and distrust in the multiple potential roles of Covid vaccines in relation to long Covid impacted perspectives on future vaccine uptake. Some participants feared being labelled as anti‐vaxx if they discussed unexpected vaccine impacts. Disengagement by healthcare professionals in discussions about the possibility of individual vaccine harms had the inverse consequence of limiting uptake of further Covid vaccines. Distrust could also grow in relation to unrealised benefits of vaccination—in this case, the official role as protection from severe infection and the unofficial role of treatment. Participants who trusted vaccines as a form of treatment struggled to access them for this use.

**Conclusion:**

The gap between scientific discourse—which recognised potential benefits *and* potential harms of vaccines in relation to long Covid—and public health discourse, which tended to focus on protection from infection, contributed to difficulties in maintaining trust after unexpected vaccine experiences. Further research to better characterise who is likely to benefit from vaccination and who might be at risk of worsening long Covid symptoms would enable better conversations between patients and healthcare professionals when making decisions about further vaccination.

**Patient or Public Contribution:**

The study was guided by a patient and public involvement and engagement (PPIE) group from project development through to dissemination. People with long Covid supported recruitment strategies, informed the development of topic guides, reviewed findings and offered suggestions for dissemination. Study participants were also invited to review and feedback on findings.

## 1 Introduction

In this paper, we explore processes of gaining and losing trust in new medical technologies, drawing on stories about unanticipated impacts of Covid‐19 vaccination in relation to experiences of long Covid. At the time of initial vaccine development in 2020, it was unclear whether Covid vaccines would improve, worsen or have no effect on the emerging post‐viral condition long Covid. In the next sections, we introduce what is currently understood from biomedical literature on Covid‐19 vaccines and long Covid, combining this with thinking from social science literature about the dynamic processes through which people develop trust in vaccines. From there, we move forward to explore how patients with long Covid made sense of unexpected vaccine impacts, analysing how this affected their trust in future Covid vaccination.

### 1.1 Long Covid and Covid‐19 Vaccination

Covid‐19 vaccines were unique in their speed of development and global deployment, building on existing coronavirus vaccine development programmes and advancements in mRNA technologies [1, 2]. They were not a singular vaccine, in that they were constituted of multiple doses, multiple brands, multiple formats designed to address different molecular and genetic targets, and successive adaptations to address evolving genetic variants of Covid‐19 [3]. While all approved UK vaccines had by definition been declared effective and safe in the prevention and control of Covid‐19 by UK regulatory authorities, there was limited research into their long‐term effects and their efficacy in other indications, notably for people already suffering from the long‐term sequelae of Covid‐19 [4].

Long Covid is a multisystem condition, variously described as post‐Covid‐19 syndrome, post‐Covid‐19 condition and post‐acute sequelae of SARS‐CoV‐2. It is characterised by many persistent symptoms affecting multiple body systems, including fatigue, headache, cognitive impairment and shortness of breath [4, 5]. The majority of people recover most or all of their functional capacity, though they can report ongoing symptoms such as fatigue [6]. Qualitative research has explored how disabling symptoms limit the ability to do things that were previously constitutive of identity, and the unpredictability of symptoms makes planning for the future difficult [7, 8, 9].

Long Covid was an emerging condition at the time of vaccine development and roll‐out. Potential impacts on long Covid were secondary to the primary purpose of the vaccines, which was protecting individuals from Covid and the serious consequences of hospitalisation and death. Multiple potential impacts of long Covid from vaccination are evidenced in the growing biomedical literature. As well as its stated purpose of reducing the severity of Covid‐19, vaccination substantially reduces (but does not entirely eliminate) the risk of developing long Covid [10, 11, 12]. For people who developed long Covid before they were vaccinated, the response to vaccination has been mixed: some people with long Covid report improvements in symptoms while others find their symptoms stay the same or worsen following vaccination [9, 10, 13, 14, 15, 16]. There is also an emerging evidence base suggesting that vaccines may cause adverse effects that share some features of long Covid, including neurological side effects [4, 17, 18]. Greenhalgh et al. [4] report that ‘rare sequelae of Covid‐19 vaccination can overlap with the clinical manifestations of long Covid, but causality has not yet been established’, advocating that global analysis of adverse event reporting and dedicated clinical and biomedical research is needed to make connections beyond correlation.

The heterogeneity in the response to vaccines in people with long Covid suggests that vaccines (of different kinds) may trigger different responses at the immunological and biochemical levels. The detailed science of variation in vaccine responses is beyond the scope of this paper (for a review, see [19]), but briefly, it is hypothesised that when mRNA or killed viral vaccines are used, a minority of people mount an excessive immune response to SARS‐CoV‐2 RNA, leading to worsening of any existing long Covid symptoms. It is further hypothesised that the new protein‐based third‐generation vaccines may avoid such reactions with long Covid, since such vaccines would avoid presenting the RNA backbone of the virus to the host's immune system. The key point for this study of people's stories about how vaccines impacted symptoms of long Covid is that there is a biologically plausible, though not yet proven, scientific explanation why some people with long Covid might develop unexpected exacerbation of symptoms from a vaccine.

### 1.2 Building and Losing Trust in Vaccination

This study seeks to contextualise the above biomedical literature within an established body of work exploring the dynamic processes by which the public gain or lose trust in vaccination. We sought to learn how perceived unexpected benefits and unexplained side effects in relation to long Covid affected trust in taking future Covid vaccines. We explore this from the perspective of people with long Covid who actively sought vaccination.

Several authors have highlighted the complexity of trust in relation to vaccination. Poltorak et al.'s [20] study of mothers' vaccine decisions in England cautioned that choosing to vaccinate does not correlate directly to trust in vaccines, or vice versa. Nurmi and Jaakola [21] aimed to nuance the category of ‘trust’ in vaccination in their study of narratives of Finnish parents who had opted out of some or all vaccinations for their children. They distinguish between mistrust—whereby people hold general suspicions in relation to institutions (such as government and the pharmaceutical industry)—and distrust—where people experience a sudden loss of trust primarily in relation to an unexpected adverse vaccine event. They argue that distrust happens quickly, but can be repaired if professionals take patient concerns seriously and if adequate medical and financial support is offered where adverse impacts are confirmed. Herzig van Wees and Strom [22], in their study of people partially or fully declining childhood vaccinations in Sweden, frame vaccine decisions as an ongoing process of enquiry rather than a fixed position. Decisions varied significantly between individuals and between vaccines: some opted for ‘partial refusal’, some ‘full refusal’ and many changed their minds over time in relation to evolving perceptions of immunity and risk. Participants in their study felt that declining vaccination in the present did not preclude vaccination in the future, and felt no connection with organised groups of ‘anti‐vaxxers’ aiming to influence others' actions.

This literature informs us that trust in vaccines as medical technologies is relational, dynamic and contextual, with considerable complexity and ambivalence in decisions among both those who take and do not take vaccines [23]. We build on existing knowledge about vaccine engagement by paying particular attention to how unanticipated vaccine experiences among people who consider themselves to ‘trust’ vaccination impacted their future vaccine intentions. Given that the biomedical knowledge described above was scant during early vaccination experiences, we explore how people developed explanations for the changes they experienced. The central question guiding this study was ‘*how do people with long Covid make sense of unexpected vaccine responses?’*. We sought to examine how participants reconciled expectations of vaccination with reported experiences, and how this shaped trust in vaccination.

## 2 Methods

### 2.1 Approach

We engaged with members of the public who experienced improvement or deterioration in long Covid symptoms, or development of new symptoms related to long Covid, following vaccination. Our ambition was to study how people accounted for gaining or losing trust in vaccines through their illness narratives. This builds on a growing body of literature examining the role of narrative in the formation of long Covid as a novel condition [7, 9, 24, 25, 26, 27]. Sociologist Arthur Frank [28] identified that illness narratives may follow different plots depending on whether the ‘trouble’ of illness is happily resolved (restitution); leads to death, disability or disfigurement (tragedy); produces some kind of fulfilment (quest); or fails to unfold coherently (chaos). For this study, our interest was in how the ‘trouble’ of long Covid was narratively connected to vaccination. This involved examining how Covid vaccines were positioned as characters in participants' stories, particularly where an unanticipated vaccine reaction represented a plot twist [29], turning a hoped‐for restitution narrative into a tragedy or chaos narrative or vice versa. We were interested in the labour of anticipating and making sense of bodily responses to vaccines [30, 31]. We paid particular attention to how the uncertainty of attribution of unexpected vaccine responses shaped trust towards vaccines as healthcare technologies. Through this approach, we considered participants' accounts of the (changing) roles vaccines played in their story. This sociologically informed study was designed to examine people's sense‐making about Covid vaccines, not to examine whether, to what extent or in whom these vaccines are effective.

### 2.2 Data Collection

This study includes 33 interviews, collected by qualitative social science researchers (including AD and AM) with people who had long Covid symptoms *and* had taken a Covid vaccine. We conducted an initial 15 interviews to collect general narratives about experiences of long Covid, including any perceived impact of vaccines (collected between May 2021 and May 2022) and a further 18 interviews with different people to explore explicit vaccine narratives (collected between November 2022 and June 2023). Sample size was guided by Malterud, Siersma, and Guassora [32] concept of ‘information power’, that is, whether a sample contains enough information to answer the research questions. For this study, this was a point where multiple different vaccine narratives were identified and enough data was available to analytically engage with the emerging research questions, with contradictions and deviant cases present but with decreasing frequency.

The study aimed for maximum variation sampling, with diversity in age, gender, ethnicity, geographical location and socio‐economic status across the United Kingdom. We recruited through GPs, long Covid clinics and community groups and also advertised through closed long Covid research and information groups on Twitter and Facebook for participants. Some participants had heard about the research through multiple routes, both online and through clinics and support groups. In the second part of the study, we aimed for balance across the sample of people who reported primarily positive or negative vaccine impacts following vaccination after the onset of long Covid. Details of the study participants are described in Table 1.

**Table 1:** Participant characteristics.

Pseudonym	Gender	Ethnicity	Age range	When interviewed	Total vaccination doses at time of interview	Reported impact of vaccine on symptoms	Reported months living with long Covid before change in symptoms
Anita	Female	Black British	50–59	May‐21	1	Negative	6
Jan	Female	White British	40–49	May‐21	2	Positive and negative	10
Michelle	Female	White British	50–59	Sep‐21	2	Positive	12
Jack	Male	White British	30–39	Sep‐21	2	Negative	0
Ruth	Female	White British	30–39	Oct‐21	2	Negative	5
John	Male	White British	60–69	Nov‐21	2	Negative	9
Muriel	Female	White British	50–59	Jan‐22	2	Negative	12
Andrew	Male	White British	50–59	Feb‐22	1	Negative	12
Sana	Female	Indian	40–49	Mar‐22	3	Positive	3
Maria	Female	Greek Cypriot	20–29	May‐22	3	Negative	12
Liz	Female	White British	40–49	May‐22	3	Negative	12
Diana	Female	British Pakistani	30–39	May‐22	3	Negative	2
Edward	Male	White British	30–39	May‐22	3	Positive	12
Surindar	Female	British Pakistani	15–19	May‐22	1	Negative	0
Jennifer	Female	White British	50–59	May‐22	4	Negative	1
Katherine	Female	White British	40–49	Jan‐23	1	Negative	12
Sam	Non‐binary	White British	40–49	Jan‐23	1	Negative	0
Clare	Female	White British	40–49	Jan‐23	6	Negative	12
Larissa	Female	White British	30–39	Jan‐23	6	Negative	12
Michaela	Female	White British	30–39	Jan‐23	1	Negative	0
Rachel	Female	White British	30–39	Feb‐23	3	Negative	2
Margaret	Female	White British	40–49	Feb‐23	4	Negative	5
Gemma	Female	White British	40–49	Feb‐23	3	Negative	8
Belinda	Female	White British	40–49	Feb‐23	3	Negative	1
Amira	Female	Indian	30–39	Mar‐23	3	Positive	11
Sophie	Female	White British	40–49	Mar‐23	3	Positive	15
Francine	Female	British Asian	30–39	Mar‐23	4	Positive and negative	2
Grace	Female	White British	40–49	Apr‐23	3	Positive	12
Paula	Female	White British	50–59	May‐23	Positive	5
Thomas	Male	White British	30‐39	May‐23	2	Negative	0
Chloe	Female	White British	30–39	May‐23	4	Positive	11
Lindsay	Female	White British	30–39	May‐23	2	Negative	12
Steve	Male	White British	60–69	Jun‐23	3	Negative	0

The interviews were conducted remotely via online video platforms or over the phone, and were audio or video recorded depending on participant preference. Interviews typically lasted 60–90 min; several were shorter in length or conducted over several sessions to accommodate participant fatigue, other long Covid symptoms, medical appointments and educational commitments. All interviews followed a similar approach, starting with an open narrative followed by some semi‐structured prompts [33]. For the general interviews, these focused on how long Covid affected their lives and reflections on experiences of seeking help. For the interviews focused on vaccines, questions explored perceptions of attribution of future vaccine intentions. Participants were given a £30 voucher to thank them for sharing their time and experiences.

Interviews were professionally transcribed, de‐identified and uploaded onto QSR NVivo 12 to support data familiarisation, coding and analysis. We mapped story structure to identify where vaccine reactions were presented as a plot twist. We approached analysis in three stages. First, AD narratively coded sections of interview text to identify the perceived ‘character’ or role vaccines played in each long Covid story, identifying vaccines as having multiple roles of ‘protector’, ‘treatment’ and ‘antagonist’. Second, AD prepared vignettes for discussion with the writing team to explore how uncertainty was managed in sense‐making about the role played by vaccines. Lastly, as a group, we undertook a thematic analysis across the corpus of narratives comparing how people recount what they had expected vaccines would do, and how the success or failure of different vaccine roles created new expectations of vaccination in the future. AD drafted an initial analytical discussion, then shared it with other authors, who provided comments, criticisms and feedback on how to refine and rethink the initial analysis. Research participants were also invited to provide feedback and comment on interim findings. Pseudonyms assigned by researchers are used throughout. Ethical approval was granted by the NRES Committee South Central ‐ Berkshire (12/SC/0495). The analysis was overseen by an independent advisory group with patient representation.

## 3 Findings

**Figure 1:**
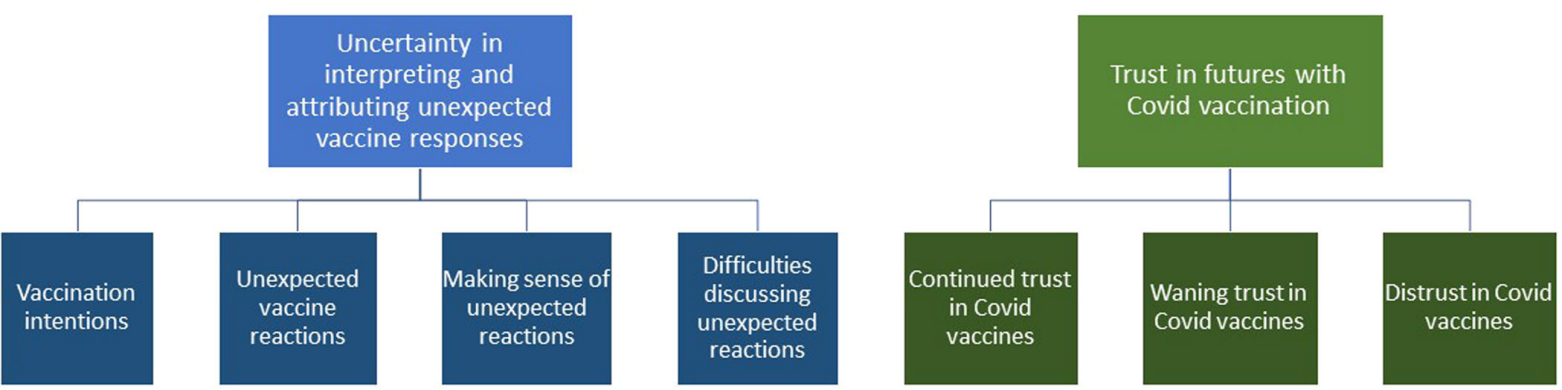
Summary of themes.

In the first section, we emphasise the uncertainty involved in attributing bodily experiences to vaccine responses, whether positive or negative. In the second section, we illustrate the complexity of maintaining trust in vaccination across both wanted and undesired impacts. The themes and sub‐themes are summarised below in Figure 1.

### 3.1 Uncertainty in Interpreting and Attributing Unexpected Vaccine Responses

#### 3.1.1 Vaccination Intentions

Participants who had long Covid at the time of their first vaccine described hearing stories in long Covid online groups that some people's symptoms had been alleviated or worsened after vaccination. Margaret, for example, discussed speculation within Facebook groups:

There are some who, who had their vaccines and they were like, ‘yeah this is, this has like cured my long Covid’, you know, or ‘this is, you know, made me terrible and I've got long Covid because of the vaccine’ and things like that, and I just thought, ‘I'll just ignore whatever they're saying and just, I am me and me will have the reaction that I'll have and I just have to deal with it, if I get better fantastic that would be excellent and if I get worse then what could be any worse at least I'll not be dead from Covid’.

Among the people we spoke with, these stories did not change their intention to get vaccinated. All participants were motivated to take a vaccine because of its primary role offering individual‐ and population‐level protection from severe Covid infection. People already living with long Covid recalled discussions that protection from further severe Covid infections could minimise the chance of their symptoms worsening. Grace, for example, said, ‘*I was just hoping it was going to stop me from getting Covid again, so that the long Covid didn't get worse.’*

#### 3.1.2 Unexpected Vaccine Reactions

For some participants, like Michelle, vaccination offered longed‐for relief from long Covid symptoms.

The first vaccine felt like going through the whole thing, my entire year. And then suddenly I thought ‘oh, hang on’. It was like the sun had come out like the cloud had gone from my brain. And I suddenly felt like myself again. I think the vaccine dealt with whatever viral remnants were continuing to aggravate my immune system.

Experiences of improvements were diverse: some described a step change in all symptoms following vaccination, with the impact of fatigue and brain fog most significant. Others felt that particular symptoms were ameliorated. Limited symptom improvements were sometimes tinged with bitter disappointment that the participant had not been, as Rachel describes, one of the ‘lucky ones’ who experienced a change in their most troubling or debilitating symptoms. Sophie noticed that a persistent rash resolved and recovered some of her sense of taste and smell. She did not experience an overall difference in her worst symptoms, although they became more predictable:

My relapses changed from 7 days on to 7 days off. And being able to actually know when the relapse was going to kick in, it just gave you a little bit of freedom to be able to actually do stuff.

For other participants, vaccination was perceived to worsen or induce new symptoms. Not being able to interpret the cause of symptom change troubled participants, as discussed by Liz:

I was getting on all right and then I had my first jab and then I believe my PoTS [postural orthostatic tachycardia syndrome] established, but I can't prove it. I can't prove it. Because it might have just happened anyway because the long Covid nurse said there are more and more people with PoTS. Especially women my age and other stuff. But I don't know.

#### 3.1.3 Making Sense of Unexpected Reactions

The strongest connection that participants could make was temporal, in that symptoms changed positively or negatively following each vaccine experience. Diana questioned her experiences ‘*I don't know if it's in my head but I do feel worse when I had the vaccine. Each time I have it, it messes me up’*. For those who felt the benefits of vaccination, difficulty in attribution limited their ability to ‘keep hold’ of physical relief from symptoms. Paula had received two vaccinations before she caught Covid and developed long Covid. She found that taking her third booster offered temporary relief from fatigue. She wondered whether she had hindered her own recovery by doing too much.

It's too much of a coincidence that it felt like this amazing ten days of feeling fabulous and probably if I had been sensible what I should have done was just rest and not do anything, you know, or just do a little bit but I just, it's like three or four months at this point of not feeling great, it was just wanting to get out and do, you know, do something, do something act normal I guess.

The absence of access to medical advice or scientific evidence about how vaccination could impact long Covid recovery made it difficult to understand changes. This uncertainty was exacerbated by the novelty of long Covid as an illness, as there was no insight as to what a ‘normal’ illness trajectory was like. Gemma, a healthcare professional who had long Covid at the point of vaccination, wondered ‘*would I have reacted anyway to the vaccine or did I only react to the vaccine because I previously had Covid?’*. Further complexity was added when participants experienced a non‐linear and uncertain journey across multiple vaccines. Chloe reflected on this:

So the first vaccine seemed to help things, the second vaccine, possibly less so. It could all be incidental, and coincidence, it's really not clear, but on the whole, I would say I have steadily been getting better since that time, so that's December, so five months ago.

It was also difficult to disentangle the impact of vaccination on recovery from other attempts to treat long Covid, further infection and the overall passage time, as described by Francine:

I feel like my story is probably quite just a bit jumbled and complicated, just because there were like multiple Covid infections, multiple boosters and all the rest of it.

Attribution was hardest when participants suspected that long Covid‐like illness had been caused by vaccination, as this role for vaccination was absent from the public conversation of potential vaccine effects at the time. Some participants hypothesised that they might have also had Covid at the time of receiving a vaccine, which caused their subsequent response. Jack described his logic:

You had Covid once. You've caught Covid again and then, at the same time, your immune system took another wallop by having the second vaccination.

Those who had greater access to detailed medical testing felt more confident in attribution. Michaela, for example, was a scientific researcher working on vaccine studies and was able to conduct tests on her own samples to develop evidence of the impact of vaccines on her immune system.

My spike antibodies: I had a … I had a reaction. I've looked at a lot of spike antibodies and the assay that we use in my workplace has a maximum threshold of 80,000 units, and I've only ever seen that in people who have had two or three vaccines plus the infection. My single AstraZeneca vaccine caused me to exceed the assay threshold within 10 days, which isn't usually a peak antibody response, and then about a month after that, my antibodies dropped to less than 400, so it was a huge immune hyper‐reaction and then it seems to be almost a … a loss of immune memory, so there was definitely something funky going on, and no evidence of infection.

#### 3.1.4 Difficulties Discussing Unexpected Reactions

Regardless of confidence in attribution, voicing potential vaccine harms was difficult. Speculating about the adverse impacts of vaccination was felt to be contradictory to public health messaging about Covid vaccine safety. This meant that some participants feared being labelled as anti‐vaxx. Sam, who suspected their symptoms began at the point of vaccination, emphasised that they were ‘pro‐vaxx and pro‐science’.

My experience of long Covid is maybe slightly different to…. I don't know what the standard experience is, if there is one, but just because mine seems to have been triggered in a different way: it wasn't triggered by catching the virus, that's as far as I know, I seem to have developed long Covid from the vac … my body's response to the vaccine, and I guess the thing I want to say before I start like talking about it, is I'm very pro‐vaxx and pro‐science and I really get that this is like an unfortunate statistic that I'm part of.

Others feared that raising the possibility of negative impacts could fuel anti‐vaxx discourse or lead them to be labelled as anti‐vaxx. Clare described this:

I suppose it's a fear that, you know, if you admit like one tiny little thing that opens the doors to all the stuff like, you know, we are, we're injecting our children with 5G so aliens can abduct them type, you know, that, that kind of like weird bizarre Covid vaccination denier, you know, sceptism.

Michaela argued against this point, reflecting that discussing vaccine impacts contributed to a vision of improved vaccine science as a whole.

There's a … a fear of being labelled antivaxx if you … if you talk about the negative vaccine side effects, and to be honest I think it's the most pro‐vaxx thing you can do because if we acknowledge the side effects we can help patients, we can improve trust, we can build safer vaccines, so if you want to be truly pro‐vaccine, you need to understand why people are reacting like this. So, I think it is the most beneficial thing we can do for vaccines is to talk about these side effects, but counterintuitively, society thinks that the worst thing we can do is talk about vaccine side effects.

Across accounts, participants contrast empirical uncertainty—drawing on notions of coincidence and luck—with embodied confidence that they had felt a distinct difference in their day‐to‐day symptoms as a result of vaccination. Participants who reported a consistent improvement or deterioration in their symptoms following vaccination felt more confident in claiming alternative roles of vaccines as treatment or antagonist of long Covid symptoms. In the next section, we examine the consequences of trust in taking future Covid vaccines.

### 3.2 Trust in Futures With Covid Vaccination

#### 3.2.1 Continued Trust in Covid Vaccines

Trust in future vaccination related to the primary role that participants attributed to vaccination and its perceived success or failure in that role. For participants who feared the deadly impacts of Covid, further vaccination was essential as a protective measure. Margaret perceived her symptoms had worsened as a result of vaccination, but had also witnessed many unvaccinated people die from Covid as an ICU nurse:

If they offered me another one [vaccine], yeah go for it because I would rather have any level of protection, whether it's 5%, 10%, 90%, 95% I would rather that than, and take the risk of a reaction or take the risk of my Covid, long Covid symptoms getting worse, or the off chance that they get better.

Even where there was suspicion that vaccination may have induced long Covid symptoms, some participants were committed to further vaccination, driven by the understanding of vaccines as delivering population‐level rather than individual protection. Steve, for example, who wondered if his first vaccine had contributed to a long Covid‐like illness, wanted to persist in vaccination.

I mean I do see my continuing to have a vaccine as maybe, you know, it's a political decision on my part of being kind of municipal about it, you know, we will all do this for the common good.

#### 3.2.2 Waning Trust in Covid Vaccines

In contrast with Margaret's and Steve's strong trust in the protective role of Covid vaccines, others reported a growing distrust in their protective capacity. Larissa, who developed long Covid following an infection in the early stages of the pandemic, was offered repeated vaccines because of a clinical vulnerability. She expressed ambivalence about future Covid vaccine intentions, in her case, because of uncertainty about their protective role. Despite being vaccinated, she was hospitalised as a result of her second infection. This led her to question the protection offered by vaccination—‘*my perception of the protection that it's giving me, and my perception of how well it works in me, has waned’*. She continued to take vaccines because ‘*the alternative could be a lot worse’*, but the security of a hoped‐for future without severe illness was shifting.

I think because I am clinically extremely vulnerable to Covid, I am in a slightly rock and hard place with the vaccines. So if they offer me another one, I will take it because I don't know what the alternative … the alternative could be a lot worse, but I am less … [erm] I think now I am less secure in the hope that it will actually really protect me from having to go into hospital. My concern now with the vaccines is more, ‘is it going to actually protect me, or am I just pumping a load of vaccine into my body?’ So, my mindset's shifted on that because I haven't got the certainty that it will actually help me.

Others simultaneously described both trust and distrust. Many people wanted further vaccination, like Sam, because of their protective qualities, but feared the consequences: ‘*it's really hard to go back to the thing [vaccination] that* … *that seems to have caused all of that [long Covid symptoms]’.* At the time of interview, Sam had taken only one dose of Covid vaccine and was continuing to shield from exposure to Covid infection: ‘*I feel completely unprotected, yeah, like I feel terrified to be totally honest’*.

Limited evidence about connections between vaccination and long Covid symptoms made seeking help to assuage distrust difficult. Unable to find professionals who would support with interpreting the cause of her initial negative vaccine reaction, Pamela's distrust deepened, and she declined further vaccine doses.

I tried to get advice from everywhere I could think of, including the vaccine hotline, and nobody gave me advice, and nobody called me back, and the only thing that I was told constantly by my doctor was that ‘there is no evidence to say you shouldn't have the vaccine’, and consequently I haven't had another vaccine.

#### 3.2.3 Distrust in Covid Vaccines

Participants who felt they could clearly attribute unwanted symptom exacerbation to vaccination articulated strong distrust. Diana had observed a clear pattern in her response to each of the three Covid vaccines she had taken, resulting in debilitating symptoms. Anticipating that this pattern would continue, she planned to avoid future vaccination:

But if my doctor now told me, ‘Oh we're going to put you in for a booster’, I'd probably run the other way. That's where I am with it. I think I'm done. I will not take it because I can see that pattern in my life.

For people who hoped vaccines would act as a treatment to alleviate long Covid symptoms, the failure of vaccines to deliver hoped‐for results could also contribute to a loss of faith in Covid vaccines. Amira declared herself recovered from long Covid after improvements following two doses of the vaccine, but later experienced a relapse after a further Covid infection. She was profoundly disappointed when her symptoms were not ameliorated by a booster. Her perception that vaccines neither protected her from long Covid nor worked consistently as a treatment generated distrust in their broader protective value.

I don't think that I have much faith that the vaccine will make it is less likely for me to get Covid or for me to have milder long Covid symptoms. I don't think that I am at risk of, you know, like death so I think the most realistic concern is debilitation and I feel like maybe it's just going to make me…. I think maybe the point I'm trying to make is that I don't see the vaccine as preventative but more as treatment.

Changing trust in Covid vaccines did not directly correlate to changing trust in other vaccines. Amira felt somewhat nervous about the impact of a flu vaccine, but in the case of new infections with no treatments ‘*like bird flu’*, she imagined herself engaging with vaccines—‘*if there's a bird flu vaccination I would take it, for sure*’. Similarly, Thomas, who was not willing to take further Covid vaccines, said:

As for how I am with vaccines now I still think I see there's a big value in some of these vaccines, so if I went to Central Africa, would I get a Yellow fever vaccine? I think I would say I probably would.

## 4 Discussion

We have examined how people affected by long Covid reconciled trust in future vaccination with unexpected responses to Covid vaccines. Participants described a range of potential roles for vaccination: as protective from further infection and as attenuating, exacerbating or inducing long Covid symptoms. They expressed differing levels of confidence in the attribution of each role, given the broader uncertainty of long Covid illness trajectories, drawing on growing expertise in interpreting their bodily experiences over time and insights gained from medical testing. Absence of medical advice or research about long Covid and vaccination was deeply frustrating for those trying to make sense of these experiences. Expressing the possibility of vaccines as causing harm was fraught, with participants worried about descriptions of adverse effects being co‐opted by ‘anti‐vaxxers’. Imagining harms was more tolerable when it was also possible to imagine a positive protective role for vaccines. Reluctance to take further Covid vaccines was connected to perception of bodily vulnerabilities, and whether they believed there would be support available if their symptoms worsened. However, these decisions were made about Covid vaccines specifically, with other considerations given to vaccines protecting against different illnesses.

Disengagement by healthcare professionals in the discussion of the possibility of individual vaccine harms had the inverse consequence of limiting uptake of further Covid vaccines for some participants. This mirrors findings from Nurmi and Jaakola [21], who identify the importance of serious engagement in potential adverse events as key to addressing distrust. We interpret that the resolutely pro‐ or anti‐vaxx polarised discourse combined to stifle the ability to voice concerns or access clinical support to interpret symptoms and make decisions about reasonable trade‐offs between potential harms and protection. This left some people in limbo, unable to determine safe routes towards vaccination or decide conclusively if they should abstain. This demonstrates a disconnect between public health discourses and the emerging scientific evidence base, which indicates a realistic possibility of improvement or worsening of long Covid symptoms following vaccination [9, 10, 14, 15, 16]. Had these stories been acknowledged earlier, they could have usefully contributed to setting directions for further research.

Moving forward from Nurmi and Jaakola's [21] work distinguishing mistrust (of institutions) and distrust (of adverse events), we also demonstrate that distrust can grow in relation to unrealised benefits of vaccination—in this case, the official role as protection from severe infection and the unofficial role of treatment—as well as unwanted negative consequences. We also demonstrate that adverse events in themselves did not unequivocally lead to distrust in vaccination. Some participants persisted in vaccination despite suspecting an impact on worsening long Covid symptoms, trading this off against hope for protection from further infection.

A central limitation of the study was that we interviewed each participant only once, meaning that we were unable to examine how vaccination intentions changed over time. We, and indeed our participants, also recognise that a narrative approach was not able to determine causality in vaccine experiences. We have called for further research using appropriate biomedical and clinical methods to assess this [4]. Nonetheless, our findings have relevance for furthering research into both long Covid and novel vaccines. The gap between scientific discourse—which recognised potential benefits *and* potential harms of vaccines in relation to long Covid—and public health discourse, which tended to focus on protection from infection, contributed to difficulties in developing coherent personal narratives about vaccine experiences and accessing support. Our participants recognised that there were unknowns about the relationship between long Covid and vaccination and wanted this uncertainty to motivate further research. While this study was sociological, not biomedical, in focus, our findings support calls from biomedical researchers for further research to better characterise who is likely to benefit from vaccination and who might be at risk of worsening long Covid symptoms. Clarity on these biological issues would enable more informed conversations between patients and healthcare professionals when making decisions about further vaccination.

Our findings have broader policy and practice implications. Building on previous research, our findings reinforce the critical importance of clinicians taking concerns about unexpected vaccine responses seriously. Distrust builds when patients feel unheard or dismissed. Most participants in this study who experienced an adverse reaction wanted to continue with Covid vaccination, but also wanted to understand the causality of their reactions so they could make an informed decision about trade‐offs of protection versus worsening of long Covid. In the context of novel vaccination campaigns, the current yellow card reporting system for adverse events is insufficient. Having dedicated mechanisms for collecting information on adverse reactions and connecting this with emerging scientific evidence is vital, as is providing a direct channel through which the public can report and seek advice on adverse events. This would relieve pressure on individual clinicians, who can offer a compassionate response to suspected adverse events but are unlikely to be able to maintain an up‐to‐date understanding of vaccine evidence during public health emergencies.

With regard to public health communication, our work offers lessons for nuancing messaging about the mechanisms of vaccine protection. Where expectations of individual protection from Covid did not appear to have been met, people were sometimes reluctant to accept boosters. It is important to balance encouragement of vaccine uptake with realistic expectations for the protection they will offer, particularly emphasising that vaccines primarily offer population rather than individual‐level protection. Another important finding was that disengagement from Covid vaccines did not necessarily preclude engagement in other forms of vaccination, which participants said they would consider on a case‐by‐case basis, reflecting the positions of participants in other studies of vaccine decliners [21, 22]. This indicates that vaccine uptake campaigns require unique messaging regarding the potential and limits of protection‐ what will motivate trust in one vaccine may not be the same for others.

## Author Contributions

**Anna Dowrick:** methodology, formal analysis, writing – original draft. **Alice Maclean:** conceptualization, methodology, formal analysis, writing – review and editing. **Sue Ziebland:** conceptualization, methodology, resources, writing – review and editing, supervision. **Trish Greenhalgh:** conceptualization, methodology, resources, writing – review and editing, supervision, funding acquisition.

## Ethics Statement

Ethical approval was provided through the normal procedures of NRES Committee South Central ‐ Berkshire (12/SC/0495).

## Consent

All interviewees gave their consent by signing a consent form.

## Conflicts of Interest

The authors declare no conflicts of interest.

## Data Availability

Data are available on reasonable request. The University of Oxford holds the copyright for the full interview transcripts and may grant data sharing permission on request.
